# Production of Modified Vaccinia Ankara Virus by Intensified Cell Cultures: A Comparison of Platform Technologies for Viral Vector Production

**DOI:** 10.1002/biot.202000024

**Published:** 2020-09-08

**Authors:** Gwendal Gränicher, Felipe Tapia, Ilona Behrendt, Ingo Jordan, Yvonne Genzel, Udo Reichl

**Affiliations:** ^1^ Max Planck Institute for Dynamics of Complex Technical Systems Bioprocess Engineering Sandtorstr. 1 Magdeburg 39106 Germany; ^2^ ProBioGen AG Goethestr. 54 Berlin 13086 Germany; ^3^ Chair for Bioprocess Engineering Otto‐von‐Guericke‐University Magdeburg Universitätsplatz 2 Magdeburg 39106 Germany

**Keywords:** continuous production, Modified Vaccinia Ankara virus, perfusion cell culture, process intensification

## Abstract

Modified Vaccinia Ankara (MVA) virus is a promising vector for vaccination against various challenging pathogens or the treatment of some types of cancers, requiring a high amount of virions per dose for vaccination and gene therapy. Upstream process intensification combining perfusion technologies, the avian suspension cell line AGE1.CR.pIX and the virus strain MVA‐CR19 is an option to obtain very high MVA yields. Here the authors compare different options for cell retention in perfusion mode using conventional stirred‐tank bioreactors. Furthermore, the authors study hollow‐fiber bioreactors and an orbital‐shaken bioreactor in perfusion mode, both available for single‐use. Productivity for the virus strain MVA‐CR19 is compared to results from batch and continuous production reported in literature. The results demonstrate that cell retention devices are only required to maximize cell concentration but not for continuous harvesting. Using a stirred‐tank bioreactor, a perfusion strategy with working volume expansion after virus infection results in the highest yields. Overall, infectious MVA virus titers of 2.1–16.5 × 10^9^ virions/mL are achieved in these intensified processes. Taken together, the study shows a novel perspective on high‐yield MVA virus production in conventional bioreactor systems linked to various cell retention devices and addresses options for process intensification including fully single‐use perfusion platforms.

## Graphical abstract

Modified Vaccinia Ankara (MVA) virus is a viral vector used for gene therapies. Here, MVA virus is produced with AGE1.CR.pIX suspension cells using different platform technologies (in batch, continuous or perfusion mode). The authors then compared and discussed the pros and cons between the different platform technologies, based on the virus production yields and other criteria such as scalability and process robustness.

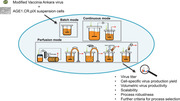

## 1 Introduction

Viral vaccines are among the most cost‐effective medical interventions^[^ ^1^ ^]^ and indispensable for control of pandemic threats.^[^ ^2^, ^3^ ^]^ However, vaccine design is complex and challenging, and viruses are often difficult to produce. Viruses are obligate parasites and require a second biological entity, a host, for replication. As one consequence, for vaccine production, naïve host cells are required at high concentrations for infection with the respective virus strain, and both (naïve and infected cell) must be amenable for biotechnological manipulation. In addition, some viruses have an extremely narrow host range and require a special or even unique cell substrate. These properties call for the design and optimization of sophisticated process trains with carefully adjusted parameters in upstream (USP) and downstream processing (DSP).

Conventional viral vaccine production relies on the production of wild‐type or attenuated viruses for vaccination.^[^ ^4^, ^5^ ^]^ In addition, there are increasing demands for viral vectors that either express surface proteins for immunization^[^ ^5^, ^6^ ^]^ or deliver foreign genomic information into their target cells for gene therapy.^[^ ^6^ ^]^ Viruses are ideal vehicles for delivery of genetic information, both for vaccine purposes and gene therapy. Issues that may affect synthetic DNA or RNA such as packaging and delivery of the payload,^[^ ^7^, ^8^ ^]^ amplification at the target site, and expression of foreign genetic information in the face of cellular defenses^[^ ^9^, ^10^ ^]^ are already inherent properties of the infectious cycle. Modified Vaccinia Ankara (MVA) virus is a promising viral vector for use against various infectious pathogens such as coronavirus,^[^ ^11^ ^]^ or for immunotherapy and the treatment of some types of cancers. MVA virus has been generated by adaptation to chicken embryo fibroblasts (CEF)^[^ ^12^ ^]^ and cannot replicate in human recipients. Because of this strong attenuation it is currently estimated that about 10^8^ infectious units per dose are required for full vaccine efficacy.^[^ ^13^ ^]^ For large vaccine programs, the necessary yields are difficult to obtain with the conventional production substrate that was also used for stepwise MVA virus attenuation: CEF cultures consist of anchorage‐dependent primary cells with a limited lifespan and require animal‐derived components for growth in culture media. The latter involves, however, the risk of contamination with adventitious agents and should be avoided for use in current good manufacturing practice production. To circumvent this concern for production of MVA virus, an avian suspension cell line (AGE1.CR.pIX) has been developed previously,^[^ ^14^ ^]^ together with chemically defined media for batch production.^[^ ^15^ ^]^ The biphasic process is initiated with a cell seed train in true suspension culture to build up high cell concentrations for infection with virus seed at the final process scale. The subsequent virus production phase involves the addition of a (also chemically‐defined) medium to induce suspended cell aggregates to facilitate virus spreading, as a large fraction of the infectious poxviruses are disseminated by direct cell‐to‐cell contact. Induction of aggregates improves virus yields and has been shown recently to enable safe and cost‐efficient production of vaccine candidates.^[^ ^16^, ^17^ ^]^ To facilitate process intensification and enable continuous virus harvesting an MVA virus strain with reduced dependency on direct cell‐to‐cell contacts would be beneficial. Such a strain, MVA‐CR19, has been obtained with help of the avian suspension cells^[^ ^18^, ^19^ ^]^ that are used in the present study.

Based on these developments, new options for process intensification were available. In this study we focused on cell cultures at high viable cell density (HCD) using fed‐batch or perfusion strategies (above 25 × 10^6^ cells mL^−1^),^[^ ^20^, ^21^ ^]^ and on continuous cultivations at low cell concentrations (chemostat; 2–5 × 10^6^ cells mL^−1^).^[^ ^22^ ^]^ High cell concentrations reached in the bioreactor through the use of perfusion systems potentially allows for higher virus titers, as long as the cell‐specific virus yield (CSVY) is kept high. In this regard, many studies have already been conducted in academic groups for virus production in perfusion cultures.^[^ ^22^, ^23^, ^24^ ^]^ More specifically, Vazquez et al. (2018) showed that AGE1.CR.pIX cells proliferate to high cell concentrations in perfusion mode using a system that consisted of a stirred‐tank bioreactor (STR) coupled to a membrane‐based alternating tangential flow (ATF) device (STR‐ATF system).^[^ ^25^ ^]^ This system led to successful production of the MVA‐CR19 virus strain.^[^ ^25^ ^]^ In addition, it was shown that pseudo perfusion in shake‐flasks (SFs) was possible and virus yields comparable to the production in the ATF system. With this scale‐down model, screening and optimization of different feeding strategies improved virus productivity.^[^ ^25^, ^26^ ^]^ A follow‐up study showed that this optimized strategy could successfully be transferred into a bioreactor set‐up leading to a hybrid perfusion process combining a perfusion strategy at HCD (>25 × 10^6^ cells mL^−1^) during the whole run with working volume (V_w_) expansion after virus infection (STR‐ATF hybrid perfusion system).^[^ ^27^ ^]^

Although a successful membrane‐based perfusion system for MVA virus production was established, various other aspects needed to be addressed to further improve productivity. The first aspect was the possibility to harvest continuously the virions released. With an average MVA virus diameter of about 450 nm, the choice of the membrane cut‐off was crucial, as most membranes were designed for removal of recombinant proteins with relatively low molecular weight. Therefore, when using ATF systems, MVA virus retention inside the bioreactor was typically the resulting scenario, even when using polysulfone (PS) and polyethersulfone (PES) hollow‐fiber (HF) membranes with pore sizes going up to 0.65 μm in perfusion mode.^[^ ^25^, ^27^ ^]^ However, it has been hypothesized that virus retention can potentially decrease the productivity through unspecific virus degradation. Hence, a continuous virus harvesting during the perfusion process should reduce virus inactivation. To test this hypothesis, we investigated two options for cell retention that are not membrane‐based, known as acoustic settler (AS) and inclined settler (IS), and coupled them to a STR (STR‐AS and STR‐IS system). Both systems were operated under optimized process parameters determined previously for influenza A virus production.^[^ ^28^, ^29^ ^]^ Compared to the membrane‐based STR‐ATF system, STR‐AS and STR‐IS systems enabled continuous virus harvesting.

The second aspect concerned the option of MVA virus production in perfusion mode using exclusively single‐use material. Considering production of MVA vectors for timely vaccine development against emerging diseases or pandemics such as COVID‐19, it would be very important to quickly set‐up high‐yield production facilities at the place of highest need. In addition, single‐use disposable components could easily be installed in laboratory containers or within hospital settings. We therefore established two different perfusion systems that are commercially available in single‐use and evaluated their performance in a few, non‐optimized scouting runs. The first system involved a single‐use orbital‐shaken bioreactor (OSB) coupled to a single‐use ATF system (OSB‐ATF system). Process conditions were adapted for MVA virus propagation in AGE1.CR.pIX cells following a previous study for influenza A virus production.^[^ ^30^ ^]^ The second system involved a small‐scale hollow‐fiber bioreactor (HFBR), again operated with process parameters based on a previous study for influenza A virus production^[^ ^31^ ^]^ allowing here, as well, MVA virus production in perfusion mode. As both studies were designed only as scouting experiments without further optimization a fair comparison of productivities is difficult. Nevertheless, these platforms are available and show a high potential for success after further optimization.

Finally, the various options for intensification of MVA virus production are compared. For reasons of comparability, we focused on AGE1.CR.pIX cell‐based processes to identify the most promising cultivation strategy. To allow for a fair comparison, virus production yields and virus titers were calculated following the same method. Overall, a whole set of process options is now available, not all fully optimized, but all with high potential to establish a large‐scale manufacturing platform for intensified MVA virus production.

## 2 Results

**Figure 1:**
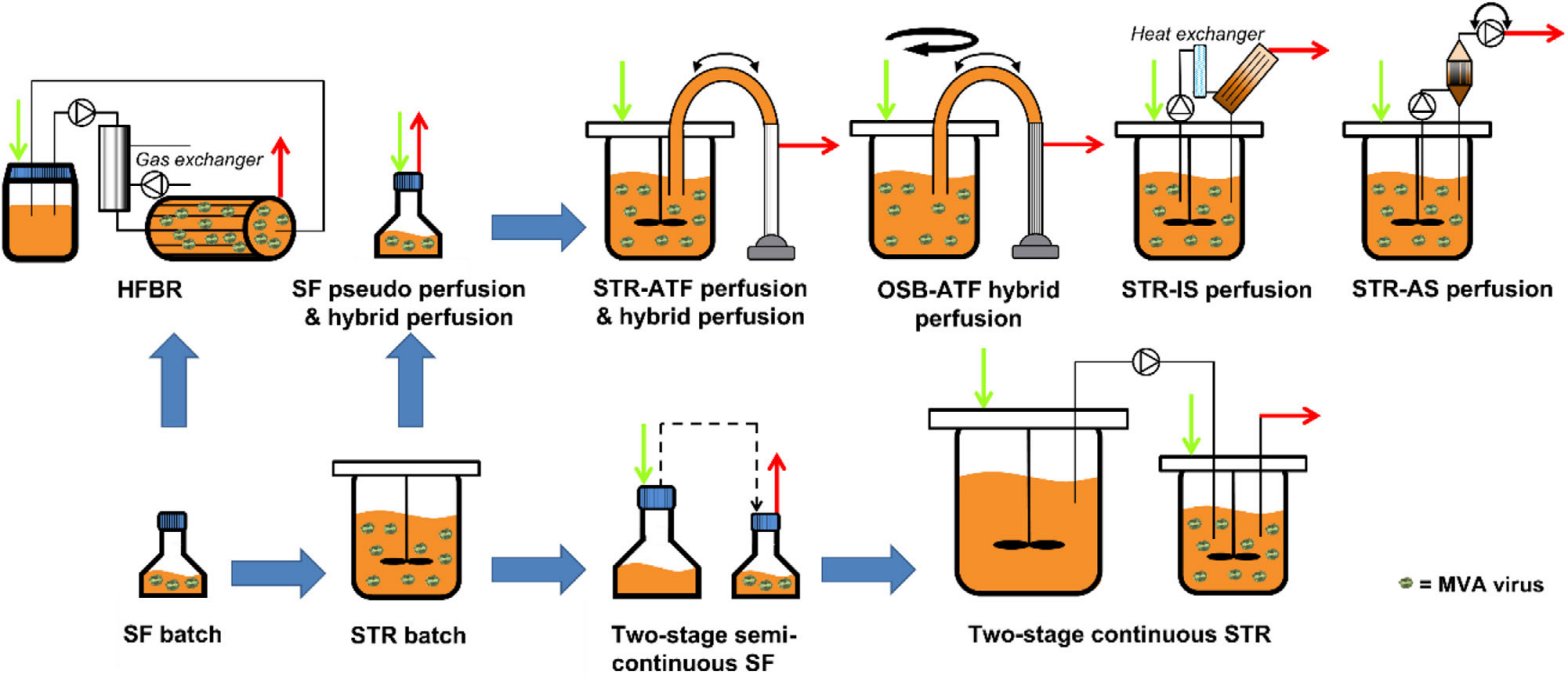
Simplified scheme of process options considered for yield comparisons towards establishment of a platform technology for MVA virus production. Green arrows indicate the continuous or semi‐continuous addition of fresh medium during cultivation. Red arrows indicate continuous or semi‐continuous removal of cell culture broth containing either only medium, medium with cell debris, medium with cells or—during virus production phase—medium with MVA virus or medium with cells, cell debris, and MVA virus. Thick blue arrows indicate stepwise process development performed in our group.

Over the last years, a considerable amount of work was already dedicated on the upstream process intensification of cell culture‐based MVA virus production. However, so far, all approaches focused on STR‐ATF systems.^[^ ^25^, ^27^ ^]^ Here, two different cell retention devices (AS and IS) were tested for continuous virus harvesting, and options for fully single‐use perfusion operation were also evaluated. Based on the newly generated data and recently published data,^[^ ^25^, ^27^, ^32^ ^]^ virus yields were calculated and compared to different intensified MVA virus production modes (**Figure** **1**) to determine the option showing the highest virus yields.

### 2.1 Cell Growth and Virus Dynamics in Perfusion Cultures with Different Cell Retention Devices

Cells were cultivated in a STR coupled to either an ATF system, an AS or an IS. To allow for a better comparison, the same cell line (AGE1.CR.pIX) with the same virus seed (MVA‐CR19.GFP) were used, and cultures were always infected at a cell concentration between 20 × 10^6^ and 30 × 10^6^ cells mL^−1^ for run 1 (STR‐ATF), run 2 and 3 (STR‐AC), and run 4 (STR‐IS). After infection, the perfusion rate was kept constant at an average of 1.65 (± 0.25) day^−1^ for runs 1–4. The process parameters selected, corresponded to optimized parameters for the specific cell retention device reported earlier for MVA virus production in perfusion mode,^[^ ^27^, ^28^, ^29^, ^33^ ^]^ see Section 4.

**Figure 2:**
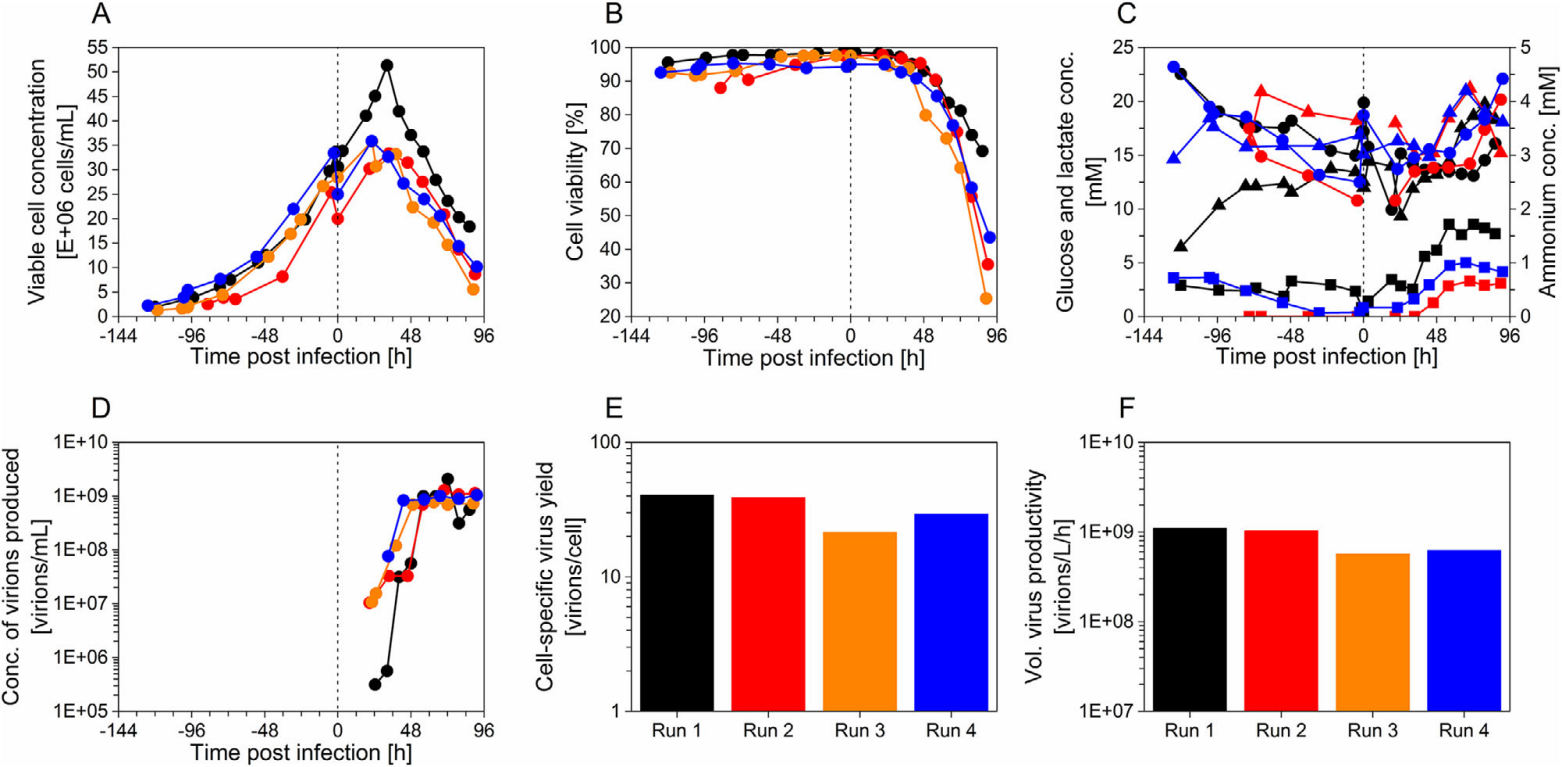
Perfusion mode cultivations in stirred‐tank bioreactor for MVA‐CR19.GFP virus production in AGE1.CR.pIX cells at high cell density. A) Viable cell concentration (●), B) cell viability (●), C) cell metabolites (glucose (●), lactate (▲), and ammonium (■)), D) concentration of virions produced (●), E) cell‐specific virus yield, and F) volumetric virus productivity of AGE1.CR.pIX cells for run 1 (ATF, black), run 2 (acoustic settler, red), run 3 (acoustic settler, orange), and run 4 (inclined settler, blue). Process parameters as described in Sections 4 and 2.1. No data available for run 3, graph C. The vertical dotted lines correspond to the time of infection.

For all three perfusion set‐ups, cell growth dynamics and cell viabilities before and after infection were compared as illustrated in **Figure** **2**A,B. In addition, glucose, lactate and ammonium concentrations are shown in Figure 2C. Similar cell growth dynamics and cell viabilities (>90%) were observed for all systems during the cell growth phase (Figure 2A,B). After MVA virus infection, the highest maximum cell concentration was observed for the STR‐ATF system compared to the STR‐AS and the STR‐IS (51 × 10^6^ cells mL^−1^ for the STR‐ATF versus 36 and 33 × 10^6^ cells mL^−1^ for the STR‐AS and STR‐IS, respectively). A decrease of cell concentrations and cell viabilities was observed about 36 h post infection (hpi) for all the three different cell retention devices (Figure 2A,B). Furthermore, no significant differences in lactate (5–20 mm) and ammonium levels (<2 mm), which are the two main by‐products of cellular metabolism, were observed. No limitation in glucose concentration (>10 mm) was observed for runs 1, 2, and 4 (Figure 2C).

To assess the performance of the different perfusion set‐ups, concentration of virions produced (C_vir, tot_), CSVY, and volumetric virus productivity (P_v_) were determined (Figure 2D–F). A similar dynamics of C_vir, tot_ was found for runs 1–4. As expected, during the first 48 hpi, low amounts of virus (<1 × 10^8^ virions mL^−1^) were detected. The maximum C_vir, tot_ was observed for all the runs between 48 and 72 hpi (Figure 2D). A similar CSVY of 20–40 virions per cell was calculated for all four runs; taking into account the error of tissue culture infectious dose 50 (TCID_50_) assays (±0.3 log_10_ (virions mL^−1^)), the differences are not significant. The same trend was observed for the P_v_, where a yield of 1 × 10^9^ virions L^−1^ day^−1^ was obtained for the ATF, again with a variation in yield for the other cell retention devices within the error of the assay (Figure 2E,F).

One concern regarding MVA virus production in STR‐ATF systems with virus accumulation inside the bioreactor is the loss of infectivity due to temperature influences, degradation due to proteases released from lysed cells, and other unspecific mechanism. Therefore, as a control, TDIC_50_ was monitored by incubating a defined amount of infectious virions in cell culture supernatant at 37 °C as described in Section 4. No decrease of MVA virus infectivity was observed for a time window of 24 h (data not shown), which corresponded to the time between the start of virus release into the supernatant and the maximum C_vir, tot_ (Figure 2D).

### 2.2 MVA Virus Production in Single‐Use Perfusion Systems—Orbital‐Shaken Bioreactor in Perfusion and Hollow‐Fiber Bioreactor

**Figure 3:**
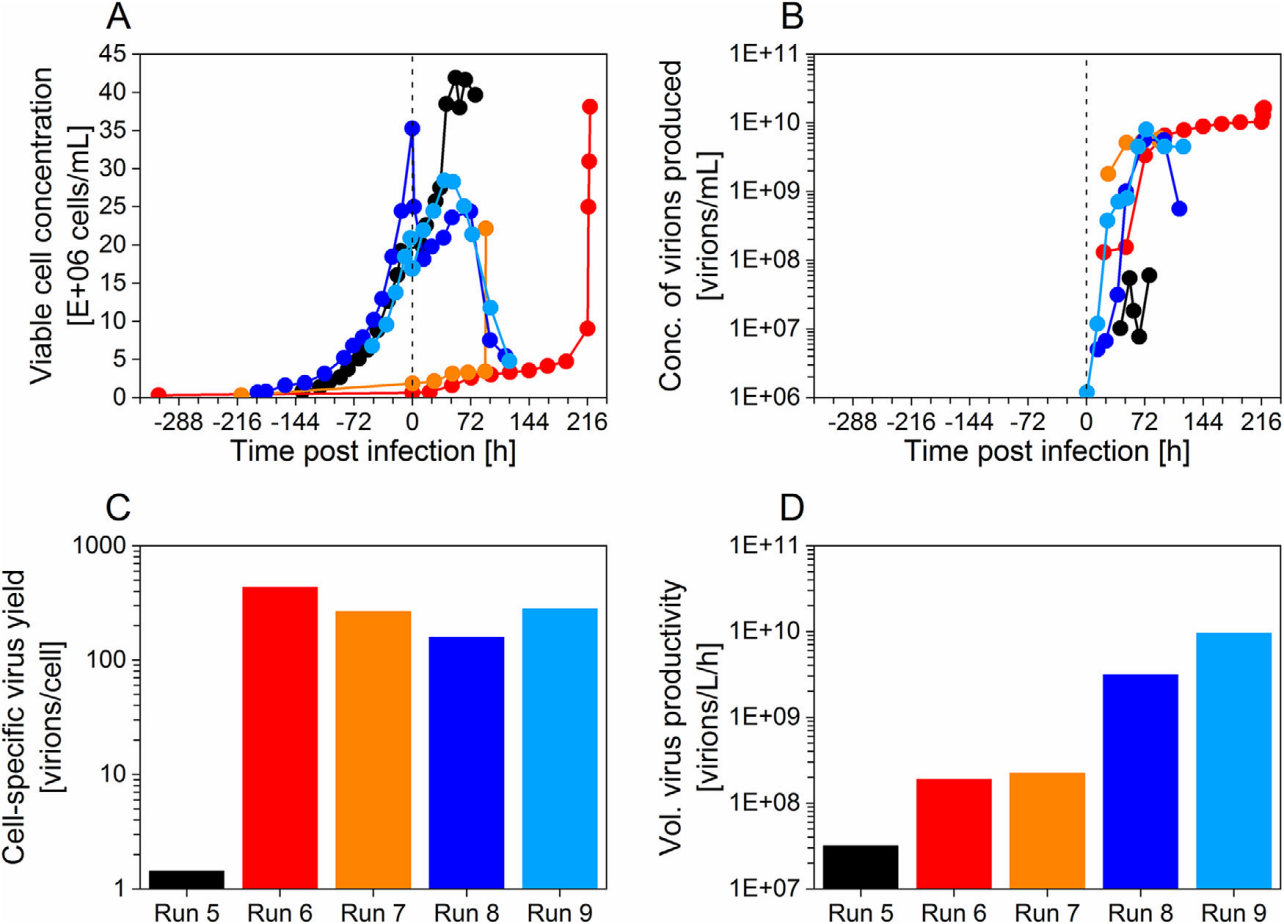
Perfusion mode cultivations in single‐use orbital‐shaken bioreactor with ATF (OSB‐ATF) and hollow‐fiber bioreactor (HFBR) for MVA‐CR19 virus production in AGE1.CR.pIX cells at high cell density. A) Viable cell concentration (●), B) concentration of virions produced (●), C) cell‐specific virus yield, and D) volumetric virus productivity for the production of MVA‐CR19 virus for run 5 (black, OSB‐ATF in hybrid perfusion mode), run 6 (red, HFBR in perfusion mode), run 7 (orange, HFBR in perfusion mode), run 8 (dark blue, stirred‐tank bioreactor with ATF in hybrid perfusion mode, control experiment, run hybrid 1 from Vazquez et al. (2019)^[^ ^27^ ^]^), and run 9 (light blue, stirred‐tank bioreactor with ATF in hybrid perfusion mode, control experiment, run hybrid 2 from Vazquez et al. (2019)^[^ ^27^ ^]^). For run 6 and 7, cell concentrations given are from samples taken from the extra‐capillary space when medium was exchanged. However, as many cells were attached to the hollow‐fiber the overall cell concentrations might be higher. For the three last sampling points of run 6 and the last sampling point of run 7, the HFBR was flushed thoroughly to detach all cells. The vertical dotted lines correspond to the time of infection.

Single‐use equipment allows for rapid and flexible virus manufacturing. In the context of process intensification, a scouting study was performed that used process knowledge from intensified influenza A virus production with single‐use equipment^[^ ^30^, ^31^ ^]^ as a proof‐of‐concept. Two different available systems were tested: the OSB (Adolf Kühner AG system) coupled to an ATF system (run 5), and the HFBR (PRIMER system from Biovest Intern. Inc.) (runs 6–7). The single‐use OSB was connected to an ATF system as no advantage regarding a virus yield, CSVY or P_v_ was observed with other cell retention devices as shown above (Figure 2D–F). Cell growth, virus titers, and yields were then compared to the two control runs reported by Vazquez et al. (2019)^[^ ^27^ ^]^) using the same cell line (AGE1.CR.pIX) and same virus strain (MVA‐CR19) (**Figure** **3**). The control runs correspond to run 8 and 9. For all cultivations, the virus was always accumulated inside of the bioreactor as the used cell retention devices are membrane‐based.

Suspension cells were infected at a cell concentration between 17 × 10^6^ and 25 × 10^6^ cells mL^−1^ for runs 5 and 8–9. Run 5 (OSB‐ATF system) reached a maximum cell concentration after infection above 40 × 10^6^ cells mL^−1^ while a lower cell concentration was obtained for the STR‐ATF system (<30 × 10^6^ cells mL^−1^, runs 8–9). However, a low maximum C_vir, tot_ of 6.0 × 10^7^ virions mL^−1^ was achieved for the OSB‐ATF compared to the STR‐ATF control runs 8–9 (5.6–8.0 × 10^9^ virions mL^−1^) (Figure 3A,B). This lead to very poor CSVY and P_v_ yields for run 5 compared to the control runs 8–9 which had a CSVY of 158–281 virions per cell and a P_v_ of 3.1–9.5  ×  10^9^ virions L^−1^ h^−1^ (Figure 3C,D).

Regarding the HFBR system (runs 6–7), cell growth dynamics differed clearly compared to the control runs 8–9. The cell concentration remained constant after infection up to 5 × 10^6^ cells mL^−1^ except at the end of the run where cell concentrations in the range 22–38 × 10^6^ cells mL^−1^ were reached due to thorough flushing of the HFBR system, detaching a large quantity of the cells adherently growing on the hollow‐fiber (HF) membrane (Figure 3A). Therefore, maximum C_vir, tot_ values of 5.9–16.5 × 10^9^ were obtained for run 6–7, which were higher than for the control runs (Figure 3B). Similar CSVYs were obtained for runs 6–7 compared to controls (Figure 3C). As the overall process time was longer compared to the control runs and, due to the high consumption of perfused media (data not shown), the P_v_ yields were about 25 times lower (Figure 3D).

### 2.3 Comparison between Different Options for MVA Virus Production in Batch, Perfusion, Hybrid Perfusion, or Continuous Mode

**Figure 4:**
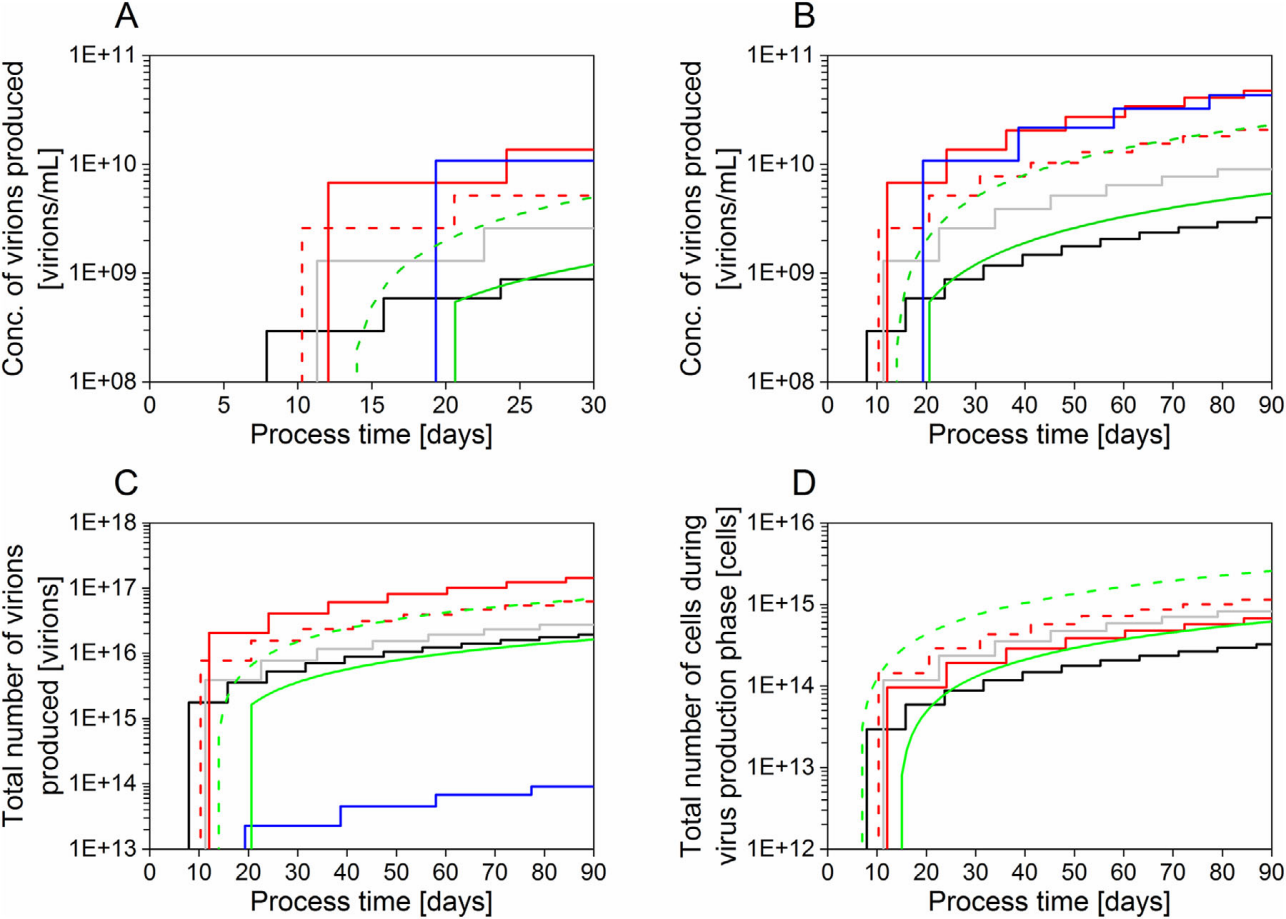
MVA virus production in batch, perfusion, hybrid perfusion or continuous mode. A) Maximum concentration of virions produced over a period of 30 days and B) over a period of 90 days. C) Total number of virions produced considering the maximum production scale for single‐use operation. D) Total number of cells present during the virus production phase considering the maximum production scale for single‐use operation. For graph D, the hollow‐fiber bioreactor (HFBR) based process was not taken into account as the monitoring of cell concentrations was difficult (Section 4). Each step corresponds to one independent run. Batch (black, average of four runs, data not shown), perfusion (grey, average of runs 1**–**4), pseudo hybrid perfusion (dotted red, average of three runs, data not shown), hybrid perfusion (red, average of runs 8–9), HFBR (blue, average of runs 6–7), two‐stage semi‐continuous shake‐flask cultivation system (dotted green, average of runs SM25‐A and SM25‐B^[^ ^32^ ^]^) and two‐stage continuous stirred‐tank cultivation system (green, run T25^[^ ^32^ ^]^). For a more accurate estimation, the process time includes 1 day for system set‐up before the start of a cell culture run and 1 day for system clean‐up/disassembly at the end of the run. For graphs C and D, the maximum single‐use production scales assumed were: batch, 6000 L;^[^ ^58^ ^]^ perfusion and hybrid perfusion, 3000 L (www.samsungbiologics.com); continuous, 6000 L (cell growth bioreactor); and 3000 L (virus production bioreactor);^[^ ^58^ ^]^ HFBR, 2.1 L.^[^ ^45^, ^46^ ^]^

**Figure** **4** shows data of this study together with results of our group^[^ ^25^, ^27^, ^32^ ^]^ obtained for other production platforms and production modes (Figure 1) to compare C_vir, tot_, total number of virions produced (Vir_tot_), and the total number of cells present during the virus infection phase.

As expected, the lowest C_vir, tot_ was achieved for MVA virus production in batch culture, although virus concentrations increased as soon as eight days post infection (Figure 4A,B). Over 90 days of virus production time, the highest C_vir, tot_ value was found for the STR‐ATF hybrid perfusion system and the HFBR (Figure 4B). Assuming that the CSVY is the same when all processes are optimized (not achieved here for continuous production), the focus would not only be on the virus production but also on the cell production capacity. What is also compared among these systems is therefore how fast and easily we could produce cells that are readily infected and deliver a product (virus), as illustrated in Figure 4D. Here, the highest theoretical amount of produced cells after infection would be obtained for the two‐stage semi‐continuous SF cultivation system (Figure 4D), if successfully transferred in large‐scale bioreactors.

Due to a limited scalability, the lowest theoretical Vir_tot_ would be obtained for the HFBR after 90 days of process time (Figure 4C). When producing MVA virus in a HFBR or in a two‐stage continuous STR cultivation system 19 and 21 days, respectively, were necessary before virus production started, which was the longest period compared to all other process options (Figure 4A–C). Compared to batch culture, a higher C_vir, tot_ was achieved after about 25 days for the two‐stage continuous STR cultivation system (Figure 4A,B). More cells could be obtained during the virus production phase with the two‐stage semi‐continuous SF system compared to a non‐continuous system after 11 days of process time (Figure 4D). Virus production in hybrid perfusion mode allowed to produce over four times more virus after 90 days of process time compared to the perfusion mode (Figure 4C). Interestingly, the small‐scale experiments (pseudo hybrid perfusion and two‐stage semi‐continuous SF cultivation) did not follow the same trends as the respective large‐scale experiments (hybrid perfusion and two‐stage continuous STR cultivation). The increase in C_vir, tot_ was twofold (after 90 day of process time) for the large‐scale hybrid perfusion system compared to the pseudo hybrid perfusion system in SFs. A fourfold decrease in C_vir, tot_ was observed when scaling‐up from the two‐stage semi‐continuous SF cultivation system to the two‐stage continuous STR cultivation system, also after 90 days of process time (Figure 4B). Furthermore, fewer cells were produced when using the two‐stage STR cultivation system compared to the semi‐continuous SF cultivation system (Figure 4D).

## 3 Discussion

In the following, the different options for operation of perfusion system characterized in this study are compared based on virus production yields. In the next step, published results for other available cell culture platforms for MVA‐CR19 virus production^[^ ^25^, ^27^, ^32^ ^]^ are taken into account using the same approach.

Surprisingly, no increase in virus productivity (based on CSVY and P_v_) was observed for the use of AS or IS cell retention devices allowing continuous harvesting (Figure 2E,F). This is in contrast to other studies on influenza A virus, lentivirus, and adenovirus production, which demonstrated advantages regarding continuous virus harvesting in perfusion cultures.^[^ ^23^, ^28^, ^29^, ^34^, ^35^, ^36^ ^]^ Regarding the cell growth phase, a similar growth dynamics and a high cell viability (>90%) was achieved. Non‐toxic levels of released metabolites (<20 mm lactate and <3 mm ammonium) were observed regardless of the cell retention device, demonstrating a healthy state of the cells allowing high productivity after infection as already observed in previous studies^[^ ^37^, ^38^, ^39^, ^40^ ^]^ (Figure 2A–C). The period for virus release in the supernatant was observed to last 24 h, between 48–72 hpi (Figure 2D). Our study also showed a high stability of the infectious MVA virus when incubated in cell culture supernatant at 37 °C for 24 h (data not shown, method described in Section 4). By consequence, for the ATF runs without an option for continuous harvesting, virus accumulation inside of the bioreactor should not have any negative impact, as long as harvesting starts about 24 h after the virus titers start to increase. This could also explain that process yields were more or less the same for processes operated with different cell retention devices. The fact that C_vir, tot_ decreased about 6.6‐fold after 72 hpi for the ATF run, still hints to a certain risk of MVA virus degradation when incubated for more extended periods (run 1, Figure 2D). As the residence time in the bioreactor was lower than 24 h due to the high perfusion rate (1.65 day^−1^), no decrease in C_vir, tot_ was observed 72 hpi for the AS or the IS based processes, which might offer a larger flexibility regarding the time of harvest compared to the ATF run (Figure 2D). In case other HF membranes will become available for use in ATF mode that allow for a continuous virus harvesting, then clearly this should be reevaluated.

The establishment of single‐use virus production systems in perfusion mode presents many advantages over traditional batch processes in stainless steel bioreactors, that is, reduced operating and validation costs coupled with the avoidance of clean‐in‐place and steam‐in‐place methods.^[^ ^41^ ^,^ ^42^ ^]^ Furthermore, single‐use concepts offer high flexibility for changes of production campaigns and installation of manufacturing facilities at the place of need in case of pandemics. So far, however, only a few studies have explored options for virus production in fully single‐use perfusion systems.^[^ ^30^, ^31^ ^]^ Similarly to Coronel et al. (2019),^[^ ^30^ ^]^ the specific growth rate of AGE1.CR.pIX cells in OSB‐ATF was equal to that in conventional STRs coupled to an ATF system. However, for the scouting experiment performed here with the OSB‐ATF system, only low titers were obtained. Furthermore, the cell concentration increased strongly after infection when using the OSB (42 vs 28 × 10^6^ cells mL^−1^). This might indicate that only a small percentage of cells was infected by the MVA virus—probably due to the non‐optimal multiplicity of infection (MOI) caused by the lack of seed virus for the chosen V_w_ of the OSB (Figure 3). This is in contrast with previous AGE1.CR.pIX cell cultivations in perfusion mode for the production of influenza A virus using the OSB where very high yields were obtained with all cells being infected.^[^ ^30^ ^]^ Nevertheless, this first scouting experiment showed that MVA virus production is possible at high cell concentrations (above 40 × 10^6^ cells mL^−1^) in single‐use systems, but that further optimization would be needed to achieve titers comparable to STR‐ATF system.

The CSVYs obtained with the HFBR seemed comparable to control runs (Figure 3C) although this value might be overestimated due to problems with cell counting. Tapia et al. (2014) estimated a theoretical MDCK cell concentration of 40 × 10^6^ cells mL^−1^ based on the known cell concentration per cm^2^ of confluent T‐flasks, and measured a cell concentration of 28 × 10^6^ cells mL^−1^ in the same HFBR system (in the same range as here: 22–38 × 10^6^ cells mL^−1^).^[^ ^31^ ^]^ A similar comparison could be performed for AGE1.CR.pIX cells. An increase of the CSVY was, however, expected in our case as adherent cells tend to allow for higher CSVY in general.^[^ ^31^ ^]^

One major difference between recombinant protein production^[^ ^20^ ^,^ ^43^ ^,^ ^44^ ^]^ and virus production in perfusion mode is that the virus cannot be produced over extended periods as virus infection involves apoptosis and cell lysis.^[^ ^22^ ^,^ ^24^ ^]^ For the establishment of a truly continuous system, two interconnected bioreactors are needed, one for cell growth and one for virus production.^[^ ^32^ ^]^ This raises the question if the footprint of a plant can also be reduced when production requires several consecutive runs in perfusion mode to harvest an equal number of virions (compared to a run in continuous mode or consecutive runs in batch). In order to evaluate the various options for virus production, not only the increase in C_vir, tot_ and Vir_tot_ over time should be considered, but also many other aspects such as process complexity or robustness, as listed in **Table** **1**.

**Table 1:** Advantages and disadvantages of MVA virus production in different cultivation systems. Each important aspect to be considered for virus production was rated from + (minimum) to ++++ (maximum)

	Batch	Pseudo perf.	STR‐ATF^a)^	STR‐AS	STR‐IS	OSB‐ATF	HFBR	Semi‐conti.	Conti.
Low complexity	++++	++	++	++	++	++	+++	+	++
Single‐use	++++	++++	++++	+	++++	++++	++++	++++	++++
High *P*_v_	+++	+++	++++	+++	+++	+	+	++	++
Process robustness^b)^	++++	++	+++	++	+++	+++	++	+	++
Small footprint^c)^	+	+++	++++	++++	+++	+	++++	+++	++++
Low manual work	++++	+	+++	+++	+++	+++	++	+	++
Scalable	++++	+	++++	+++	+++	++++	++	+	++++

Pseudo perf., pseudo perfusion in shake‐flask; STR, stirred‐tank bioreactor; AS, acoustic settler; IS, inclined settler; OSB, single‐use orbital‐shaken bioreactor; semi‐conti., two‐stage semi‐continuous shake‐flask cultivation system; conti., two‐stage continuous stirred‐tank bioreactor cultivation system.

^a)^ Includes the virus production in perfusion mode and in hybrid perfusion mode;

^b)^ Estimated risk of failure during the run;

^c)^ Takes into account the working volume of the bioreactor plus the volume of the cell retention device coupled to the bioreactor to produce MVA virus in perfusion mode (corresponds to the Cvir, tot data presented in Figure 4A,B).

Batch cultures and cultivations in the STR‐ATF system show most advantages regarding MVA virus production as illustrated in Table 1. The biggest advantages of the batch production over the other manufacturing platforms are the low complexity of the process, high process robustness, and the low demand for manual work (Table 1). However, one major drawback is the relatively large footprint needed to produce equal number of virions compared to any other processes, as also shown in Figure 4C.

Cultivations in HFBR could be an interesting option for process intensification. Higher C_vir, tot_ compared to the batch, perfusion, and continuous systems were obtained with the HFBR, making this production platform one of the best options in terms of C_vir, tot_ after 19 days (Figure 4A,B). However in contrast to the STR‐ATF system, it is not easily scalable due to challenges regarding uniform cell seeding for large surfaces (unpublished results). HFBRs with up to 10 times the membrane area (2.1 m^2^, AcuSyst‐Xcellerator, Cell Culture Company) are available.^[^ ^45^, ^46^ ^]^ This is, however, only a scale‐up factor 42 compared to the tested HFBR (Figure 4C), and limits MVA virus production to laboratory scale use and production of material for first clinical trials. Furthermore, the HFBR cultivation resulted in low P_v_ values (Table 1 and Figure 3D), which could be explained by the high consumption of medium to produce large amounts of virions. However, this could be taken into account if media consumption would be the limiting cost factor.

A major drawback of two‐stage continuous STR cultivation systems, as shown in Table 1, is their low robustness due to relatively high equipment requirements and complex handling. Furthermore, extended process times bear a certain risk of unwanted virus mutations, although this was not reported for MVA virus.^[^ ^32^ ^]^ Overall, the process time has to exceed 25 days before this option outperforms batch processes (Figure 4A,B). However, continuous mode operation also has the potential for significant scale‐up in volume and small‐scale semi‐continuous experiments showed not only high Vir_tot_ but also the highest increase in cell number during the infection phase (Figure 4C,D). Accordingly, it has the potential to reach the highest virus yields after 11 days in case that the CSVY would be in a similar range as for the other optimized processes. However, it has to be taken into account that *C*_vir, tot_ of the two‐stage continuous STR cultivation system was reduced threefold because of the large V_w_ needed for the cell growth plus the virus propagation phase. As batch cultures in SFs are the performance benchmark for optimizing larger batch processes in bioreactors, two‐stage semi‐continuous SF cultivation should function as a performance benchmark for optimizing a two‐stage continuous STR cultivation.

A difference was observed after scale‐up from SFs to STRs for pseudo perfusion and semi‐continuous experiments. Cultivations in SFs show several significant differences compared to STRs, that is, the ratio between the maximum local energy dissipation and the mean power input is smaller. However, optimization of these process options in parallel (mini)bioreactors could circumvent scale‐up issues.^[^ ^47^, ^48^, ^49^, ^50^ ^]^

Cultivations using the OSB‐ATF system resulted in rather low production yields (Figure 3C,D). Furthermore, this set‐up required a relatively large footprint (Table 1) which makes this option less attractive. Eventually, the use of an IS or an AS device would also be an interesting option compared to the ATF system; however, AS devices are at the moment not available in single‐use and the IS has a rather large footprint (Table 1).

A limitation of our approach is the limited number of cultivations performed for each set‐up. As it is often the case for complex biotechnological processes, reproducibility can be an issue. In addition, due to the rather large error of infectivity assays used for virus quantification (Section 4), differences in P_v_, Vir_tot_, or CSVY have to be rather large to be considered significant. Accordingly, our investigation (and other similar studies on virus production) can only serve as a first orientation regarding the set‐up of process platforms for large‐scale manufacturing. Nevertheless, it can serve as a first orientation regarding the pros and cons of a large number of process options, and provide a solid basis for a more detailed process intensification approach.

Another limitation of the present study is the lack of a cost‐effectiveness analysis when evaluating the different process options. Various studies have used static or dynamic models to compare costs of fed‐batch and perfusion strategies in recombinant protein production.^[^ ^51^, ^52^, ^53^, ^54^ ^]^ However, to our knowledge, no such study was performed for virus production, so far. Also, besides large‐scale production of MVA virus based vaccines, smaller scale applications in gene therapy should be considered in future studies. Software like SuperPro Designer (Intelligen Inc., USA) could support this and allow to identify key factors limiting the use of particular production systems. Finally, for a complete analysis, DSP should also be integrated, which would further increase the level of complexity.

Intensified USP with increased MVA virus titers can have a significant impact on the DSP. Depending on unit operations established for conventional MVA virus‐based processes, it might be required to modify DSP, that is, cell clarification and product concentration steps before chromatography‐based operations are used. In fact, DSP efficiency might be the criterion for the selection of the appropriate USP platform for an end‐to‐end virus production process. For example, lower yields in virus clarification or a higher number of DSP unit operations might be needed, potentially reducing the overall production yield and the economical attractiveness of the selected USP production platform. A recent study evaluating different production platforms for adeno‐associated virus estimated DSP costs to be equal to about 40% of the overall cost of good per dose for a 1000 L suspension cell culture in batch.^[^ ^55^ ^]^ However, in this case, neither the DSP technique and DSP yield nor DSP costs per dose were influenced by the intensification of the USP. Thus, the overall impact of DSP on the economical modelling was considered to be negligible.^[^ ^55^ ^]^

Scouting experiments performed by our group did not result in a significant decrease in yields for the purification of MVA virus from STR‐AS perfusion harvests or STR batch cultivations using steric exclusion chromatography (data not shown). However, a recent study of our group indicated possible issues with influenza A virus production in a STR‐ATF system where higher titers and product accumulation in the bioreactor resulted in the formation of large virus aggregates in contrast to a STR‐AS perfusion system allowing continuous harvesting.^[^ ^28^ ^]^ In particular, the presence of such large‐sized aggregates might impair clarification and subsequent chromatography steps developed for normal batch USP conditions. As dilution by continuous harvesting decreases proteins, host‐cell DNA and the virus concentration in the medium, STR‐AS perfusion and STR‐IS perfusion might facilitate DSP in general. For the two‐stage continuous STR, the purification yield is expected to be similar to batch STR as similar virus titers are obtained in the harvest.^[^ ^32^ ^]^ Regarding the amount of process‐related impurities per produced dose, it was observed for influenza A virus production that the amount of host‐cell DNA and total protein per virions was not changed when producing the virus with a STR batch, a STR‐ATF perfusion, or a STR‐AS perfusion.^[^ ^28^, ^33^ ^]^ Further studies would be needed to tackle the purification processing following intensified USP.

In conclusion, we compared various options for MVA virus production in batch and perfusion mode using three different cell retention devices. Virus yields did not depend on continuous harvesting due to the high stability of infectious MVA virus titers at 37 °C. However, continuous harvesting may offer more flexibility regarding the time of harvest. Scouting experiments with a HFBR or an OSB‐ATF system indicate that establishment of a fully single‐use perfusion process should also be considered. However, regarding the OSB‐ATF system, further optimization would be needed to increase productivity. Finally, a broad comparison between all available process options indicated an advantage of hybrid perfusion in a STR‐ATF system over standard perfusion and continuous cultivation regarding footprint reduction, P_v_, scalability, process robustness, and options for single‐use manufacturing. Once optimized, however, a two‐stage continuous STR cultivation system might offer the highest potential for production of large volumes as it allowed to achieve the highest number of cells during the infection phase, which is necessary for obtaining maximum virus yields. Batch processes have still various advantages compared to cultivations performed in STR‐ATF systems (Table 1). However, there is a higher potential for the hybrid perfusion STR‐ATF to outperform batch processes if advantageous aspects of batch processes (such as lower complexity, high robustness, and lower demand for routine tasks and supervision) can also be improved for perfusion processes. With the presented process options, several high‐yield platforms are now available to choose for efficient and economic viral vector manufacturing for future needs.

## 4 Experimental Section

### Virus and Cell Line

The virus strains MVA‐CR19 and MVA‐CR19.GFP were provided by ProBioGen AG (Germany). For virus propagation, the immortalized avian suspension cell line AGE1.CR.pIX (ProBioGen AG, Germany) was cultivated in CD‐U3 medium (Biochrom‐Merck, Germany) supplemented with 2 mm L‐glutamine (Sigma, Germany), 2 mm L‐alanine (Sigma, Germany), and 10 ng mL^−1^ recombinant insulin‐like growth factor (LONG‐R^3^ IGF, Sigma, Germany).

### Analytics and Yields Calculations

Viable suspension cell concentration and percentage viability were determined using a Vi‐CELL XR (Beckman‐Coulter, USA). Cells in the HFBR system, not growing in true suspension, were counted as described earlier.^[^ ^31^ ^]^ Glucose, glutamine, lactate, and ammonium concentrations were measured using a Bioprofile 100 plus (Nova biomedical, USA).

For virus titration of the MVA‐CR19.GFP virus strain, a TCID_50_ assay was performed as described by Nikolay et al. (2020)^[^ ^26^ ^]^ with a standard deviation of ± 0.3 log_10_(virions mL^−1^). For virus titration of the MVA‐CR19 virus strain, the TCID_50_ was performed as previously described with a standard deviation of ± 0.4 log_10_(virions mL^−1^).^[^ ^27^ ^]^ TheP_v_ in virions L^−1^ day^−1^ and the CSVY in virions per cell were calculated as described previously by Granicher et al. (2020).^[^ ^28^ ^]^ For the specific case of the HFBR system, the CSVY was calculated taking into account the total number of cells produced as described previously for influenza A virus production with MDCK cells.^[^ ^31^ ^]^ The concentration of infectious MVA virions produced (C_vir, tot_, virions mL^−1^) was calculated as follows:

(1) $$C_{\mathrm{vir},\;\mathrm{tot}}\;\left[\mathrm{virions}\;\times \;mL^{-1}\right]\;=\frac{\sum \mathrm{Vi}r_{\mathrm{tot}}\;\left[\mathrm{virions}\right]}{V_{w}\;\left[\mathrm{mL}\right]}\;$$

With Vir_tot_, the total number of virions produced in the harvest vessel and in the bioreactor (if required cumulated over several consecutive runs using the same bioreactor) as described by Granicher et al. (2020)^[^ ^28^ ^]^ and V_w_, the working volume. For continuous processes, Vir_tot_ values were added up over time. Note, on a linear scale and considering only the error of the TCID_50_ assay for the MVA‐CR19.GFP virus strain, an error of 100% for the upper value and 50% for the lower value has to be taken into account for C_vir, tot_, Vir_tot_, CSVY, and P_v_.

### Modified Vaccinia Ankara Virus Production in Batch Mode

For an optimal MVA virus production in batch mode, a strategy described previously was used.^[^ ^56^, ^57^ ^]^ Briefly, cells were cultivated until a cell concentration of 4 × 10^6^ cells mL^−1^ was reached. The cell culture was then diluted by a factor of two with fresh medium and infected at a MOI of 0.05 virions per cell. The virus was treated before infection in a sonication water bath for 1 min at 45 kHz to avoid aggregate formation.^[^ ^26^ ^]^

### Intensified Perfusion Systems

Cells were cultivated to a viable cell concentration of 25–50 × 10^6^ cells mL^−1^. During the cell growth phase, a constant cell‐specific perfusion rate of 0.06 nL cell^−1^ day^−1^ was manually adjusted via the perfusion rate (reactor volume per day, in day^−1^).^[^ ^26^ ^]^ At the time of infection, the cell culture medium was renewed with fresh cell culture medium by increasing the perfusion flow rate for 3 h before infection. As for batch processes, an MOI of 0.05 was used with a sonicated seed virus, except for the large‐scale OSB‐ATF system. Due to seed virus limitation, an MOI of 0.01 was used in this case.

For small‐scale pseudo perfusion cultivations in HCD, baffled SFs with 50 mL V_w_ (Thermo Fisher Scientific, USA) were used as described previously.^[^ ^26^ ^]^ The cell culture was centrifuged each time and the cell culture free supernatant was removed and replaced with fresh CD‐U3 medium.

For membrane‐based perfusion, an ATF2 system (Repligen, USA) was used as described previously for MVA virus production.^[^ ^25^, ^33^ ^]^ A polyethylene sulfone HF membrane (0.2 μm pore size, Spectrum, USA) was used for the ATF mode. The exchanged volume flow rate was equal to 0.9 L min^−1^. When producing the MVA virus with an AS (10 L acoustic chamber version, SonoSep Technologies, Austria), perfusion was performed as previously described for influenza A virus production in perfusion mode.^[^ ^28^ ^]^ An acoustic power of 3 W was applied for efficient cell retention. In another setting, an IS (CS10, Biotechnology Solutions, USA) was used as described by Coronel et al. (2020).^[^ ^29^ ^]^ The IS was operated at a recirculation rate of 35 mL min^−1^, intermittent vibration (15 s on, 10 min off) and with 30° angle. Water at a temperature of 27 °C was recirculated for heat exchange. The ATF, AS, or IS cell retention devices were coupled to a 1 L STR (BIOSTAT, Sartorius, Germany). Settings for cultivations were pH 7.2, dissolved oxygen (DO) 40% and 120–180 rpm (pitched blade impeller). DO and pH were controlled by sparging of O_2_ and CO_2_, respectively.

For the scouting experiments regarding single‐use perfusion bioprocesses at laboratory scale, the OSB‐ATF system (maximum V_w_: 10 L, Adolf Kühner AG) was operated as described previously for HCD influenza A virus production.^[^ ^30^ ^]^ For perfusion operation, an ATF2 controller (Repligen) together with a single‐use PES HF membrane (0.2 μm pore size, Refine) was used. In order to maximize productivity, a hybrid perfusion mode approach was adopted as described by Vazquez et al. (2019).^[^ ^27^ ^]^ Briefly, the V_w_ was increased from 5000 to 6300 mL during the infection phase while keeping the perfusion at steady‐state. In a second set‐up, a HFBR (PRIMER HF, 50 mL V_w_, 0.5 m^2^, Biovest International Inc., USA) was used and operated as described previously by Tapia et al. (2014)^[^ ^31^ ^]^ for influenza A virus production. Medium was recirculated constantly in the HFBR and multiple virus harvests inside of the bioreactor were performed. Temperature was kept at 37 °C, and the pH was monitored and manually controlled between 7.2 and 7.4.

To investigate the MVA virus stability, cell culture supernatant with an initial virus titer of 3.06 × 10^7^ virions mL^−1^ was incubated in 1.5 mL vials in an incubator at 37 °C and kept at a pH over 7.2 (not shaken). The TCID_50_ titer was measured after 6, 12, and 24 h, in triplicate.

## Conflict of Interest

G.G., F.T., I.B., Y.G., and U.R. declare that they have no conflict of interest. I.J. is an employee of ProBioGen AG which has established the AGE1.CR.pIX cell line and developed the MVA‐CR19 virus strain.

## Author Contributions

GG, FT, IJ, YG and UR contributed conception and design of the study. GG, FT and IB performed the experiments. GG, FT and YG analyzed the data. GG, IJ and YG wrote the manuscript. All authors contributed to manuscript revision, read, and approved the submitted version.
